# Effects of ergothioneine-enriched mushroom extract on oxidative stability, volatile compounds and sensory quality of emulsified sausage

**DOI:** 10.5713/ab.20.0817

**Published:** 2021-03-11

**Authors:** Ye Tao, Shan Xiao, Jiaming Cai, Jihui Wang, Lin Li

**Affiliations:** 1 Engineering Research Center of Health Food Design & Nutrition Regulation, School of Chemical Engineering and Energy Technology, Dongguan University of Technology, Dongguan 523808, China; 2 College of Biological Engineering, Dalian Polytechnic University, Dalian 116034, China

**Keywords:** Ergothioneine, Mushroom Extract, Oxidation Stability, Sensory Evaluation, Volatile Compounds

## Abstract

**Objective:**

The aim of this work was to assess the effect of ergothioneine (ESH)-enriched mushroom extract on oxidative stability, volatile compounds, and sensory quality of emulsified sausage.

**Methods:**

The ESH content was determined by high performance liquid chromatography. The antioxidant activity of *Flammulina velutipes* (*F. velutipes*) extract was determined through radical-scavenging activity of 1,1 diphenyl-2-picryl-hydrazyl, 2,2-azinobis (3-ethylbenzothiazoline-6-sulfonic acid) and hydroxyl radicals. Four different groups of emulsified sausage were manufactured: control, no antioxidants; BHA, 0.01% butylated hydroxyanisole; EEME, 0.8% ESH-enriched mushroom (*F. velutipes*) extract; AE, 0.012% authentic ESH, after storage for 14 days (at 4°C), the quality of sausage including oxidative stability (2-thiobarbituric acid reactive substances and protein carbonyls content), volatile compounds and sensory quality were studied.

**Results:**

It was demonstrated that adding ESH-enriched *F. velutipes* extract to sausage could effectively prevent lipid and protein oxidation, and its efficacy was equivalent with 0.01% BHA. During meat processing, the ESH mainly contributed to the antioxidative activity of *F. velutipes* extract. The flavor and sensory attributes of emulsified sausage were improved through adding ESH-enriched *F. velutipes* extract.

**Conclusion:**

Accordingly, the extract of *F. velutipes* contained high-level of ESH and could be a good antioxidant candidate for processed meat production.

## INTRODUCTION

**Figure 1:**
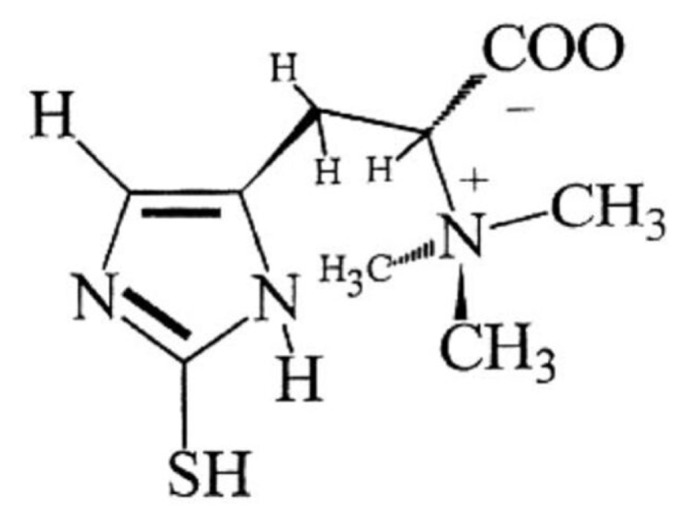
The structure of ergothioneine (ESH).

The ready-to-eat (RTE) meat products are popular for consumers all over the world, because they are convenient, time-saving, tasty and nourishing. While, the shelf-life of RTE meat products is generally short, and oxidation reaction is one of the main causes of quality deterioration [1]. To reduce the oxidation level of RTE meat products, synthetic antioxidants such as butylated hydroxytoluene (BHT), butylated hydroxyanisole (BHA) and tertiary butylhydroquinone (TBHQ), as well as nitrite and nitrate are extensively used to prevent the occurrence of oxidation reaction and further extend shelf-life of RTE meat products [1,2]. While, as consumers increased their interest in purchasing natural products, more and more researchers began looking for novel and natural antioxidants for developing “clean label” meat products [2]. Recently, ergothioneine [3] (ESH, 2-mercaptohistidine betaine, Figure 1) attracted considerable interest from researchers because of its strong antioxidant property. Many *in vitro* studies have proved ESH to be an effective natural antioxidant. As described by Franzoni et al [4], compared to classic antioxidants glutathione (GSH), uric acid and trolox (6-hydroxy-2,5,7,8-tetramethylchroman-2-carboxylic acid), ESH was the most active scavenger of free radicals. Stoffels et al [5] demonstrated that ^1^O_2_ significantly favored ESH over GSH more than 50-fold for the initial reaction. Dong et al [6] indicated that addition of only 20 and 100 μM ESH could effectively inhibit alloxan-induced lipid peroxidation of phosphatidylcholine liposomes by 67% and 100%, which was more than twice the protective effect of coenzyme Q10. Pahila et al [7] demonstrated that the 1,1 diphenyl-2-picryl-hydrazyl (DPPH) radical scavenging half maximal effective concentration (IC50) of ESH could compete with ascorbic acid, and ESH revealed a stronger scavenging capacity for oxygen radicals than ascorbic acid and GSH.

Early research showed that mushrooms contained higher levels of ESH than other dietary sources [8]. Reports indicated that ESH was naturally present in various genera of mushrooms, the levels ranged from 0.15 to 7.27 mg/g dry weight, and the highest concentration was observed in yellow oyster and porcini [9]. Since purification of ESH from mushroom is expensive and time-consuming it cannot meet the requirements of practical application, therefore, many studies focused on application of ESH-enriched mushroom extract to keep muscle food fresh. Based on the study reported by Bao et al [9], regarding lipid oxidation occurring in tuna meat, addition of 5 mL of mushroom extract to 100 g of minced bigeye tuna meat was more effective than adding ascorbic acid sodium salt (0.05%) or α-tocopherol (0.05%). Bao et al [10] demonstrated that the hydrophilic extract from the mushroom’s fruiting body was rich in ESH and had the ability to control the oxidation of lipid and myoglobin in homogenates yellowtail dark muscle. Researchers also concluded that ESH-enriched mushroom extract was effective for controlling melanosis in crab [11] and shrimp [12], they pointed out that ESH-enriched mushroom extract could be a novel and effective alternative to synthetic melanosis-inhibiting agents. Pahila et al [13] indicated that addition of ESH-enriched mushroom extract could successfully control lipid oxidation and discoloration in salmon muscle stored at low temperatures. Based on these studies, to our knowledge, only few studies focused on application of ESH-rich mushroom extract in processed meat, and in food matrix, whether ESH in *Flammulina velutipes* (*F. velutipes*) extract plays a major role in antioxidation is still unknown. Therefore, in this study, the effects of ESH-enriched mushroom extract on oxidative stability, volatile compounds and sensory of emulsified sausage were detected. Meanwhile, a comparative study on the antioxidant activity in ESH-enriched mushroom extract, BHA and authentic ESH was conducted.

## MATERIALS AND METHODS

### Mushroom extract preparation

The cultivated mushrooms *F. velutipes* were purchased from a local market in Dalian, China. The fruiting bodies of mushrooms were freeze-dried and ground to a fine powder using a food processor (YR-500A, Ying Run Industrial Co., Jinan, China). The mushroom powders were stored at −20°C until further analysis.

Mushroom extracting was carried out in triplicate for each sample. A 10 g of the *F. velutipes* powder was extracted by adding 580 mL of 70% (v/v) ethanol, followed by water bath for 15 min at 53°C, then centrifuged (3–30KS, Sigma, Osterode am Harz, Germany) at 11,363 g for 10 min at 4°C to obtain a supernatant, and then evaporated to a quarter of the original volume in *vacuo*, then added 95% (v/v) ethanol to the original volume and evaporated to dryness using a vacuum rotary evaporator (Scientz-100F, Biocool Co., Beijing, China).

### Determination of ergothioneine content

The ESH content was determined using the method as described by Lee et al [14] with some modifications. Dissolved 1 g mushroom extract in 100 mL water, after passing through a 0.22 μm microporous membrane, this was injected into the HPLC (E2695, Waters, Milford, MA, USA) equipped with Hilic column (4.6 mm×250 mm×5 μm, Agilent Technologies, Palo Alto, CA, USA). The mobile phase was water: acetonitrile = 3:97, a flow rate of 1.0 mL/min was used, injection volume was 10 μL. The ESH level was quantified by monitoring absorbance at 254 nm with an ultraviolet (UV) detector, comparing the standard curve obtained from the standard ESH (purity ≥98%, Sigma, St Louis, MO, USA). The concentration of ESH in *F. velutipes* extract was 15.29±0.52 mg/g extract.

### Determination of free radical scavenging ability

One milligram *F. velutipes* extract was diluted with distilled water to 100 mL for next determination.

#### 1,1 Diphenyl-2-picryl-hydrazyl radical scavenging activity

The DPPH radical scavenging activity was determined according to Zhang et al [15] with some modifications. Three milliliters of 100 μmol/L DPPH-methanol solution was mixed with 1 mL mushroom extract, and the tubes were vigorously shaken 30 s for mixture and incubated for 30 min in the dark. Then the corresponding absorbance (A1) at 517 nm and absorbance of the samples mixed with methanol (A2) were read. In addition, the absorbance (A0) of the control (DPPH solution without samples) at 517 nm was also recorded. The scavenging rate was calculated by the following equation:

$$\text{DPPH radical scavenging activity}(\%)=\left(1-\frac{\text{A}1-\text{A}2}{\text{A}0}\right)\times 100$$

#### Hydroxyl radical scavenging activity

The hydroxyl radical scavenging activity was measured according to the method described by Zhou et al [16]. Firstly, 2 mL of mushroom extract and ascorbic acid were mixed with 0.6 mL of 6 mM salicylic acid-ethanol solution, 2 mL of 6 mM FeSO_4_∙7H_2_O aqueous solution and 1.4 mL of 6 mM H_2_O_2_. After thoroughly mixed, they were incubated in a water bath at 37°C for 30 min. The absorbances of the mixture with (A1) or without salicylic acid (A2) were recorded at 532 nm. Meanwhile, the absorbance of the control without samples (A0) at 532 nm was recorded. The scavenging rate was obtained using the following equation:

$$\text{Hydroxyl radical scavenging activity}(\%)=\left(1-\frac{\text{A}1-\text{A}2}{\text{A}0}\right)\times 100$$

#### 2,2-azinobis (3-ethylbenzothiazoline-6-sulfonic acid) radical scavenging activity

The 2,2-azinobis (3-ethylbenzothiazoline-6-sulfonic acid) (ABTS) radical scavenging activity was determined following the method described by Thaipong et al [17] with minor modifications. Firstly, the ABTS stock solution was prepared, and then the solution was prepared into work solution with 50% ethanol until diluted to the absorbance of 0.70±0.02 units at 734 nm. And then, 3.9 mL ABTS work solution was mixed with 0.1 mL mushroom extract, and the tubes were vortexed for 45 s, reacted at 25°C for 7 min. Then the absorbances of samples mixed with extract (A1) and water (A2) were read at 734 nm. Meanwhile, the control (ABTS solution mixed with 70% ethanol) (A0) at 734 nm was also recorded. The scavenging rate was calculated by the following equation:

$$\text{ABTS radical scavenging acitivity}(\%)=\left(1-\frac{\text{A}1-\text{A}2}{\text{A}0}\right)\times 100$$

### Emulsified sausages preparation

Four different groups of emulsified sausage were manufactured: control group, no antioxidant; EEME group, added 0.8% ESH-enriched *F. velutipes* extract; AE group, added 0.012% standard ESH, and this concentration was same as the ESH content in mushroom extract; BHA group, added 0.01% BHA. The pork lean and back fat were separately ground through a 12-mm plate. Sausages were prepared with pork lean (80%), back fat (20%), and the following additives (g/kg): NaCl (25), sugar (50), soy sauce (15), liquor (10, alcohol degree 52%), ground pepper (1), sodium glutamate (0.2). The mixture was chopped to obtain a homogeneous mass with a ZB-5 bowl chopper (Huaying Food Machine Co., Hebei, China), and marinated for 10 h at 4°C. And then, the meat batter poured into the pig casing at the temperature of 4°C. The diameter of the sausage casing was about 2 cm. All sausage batches were boiled in water until the central temperature of final products attained to 72°C±2°C, and then they were cooled to room temperature, packed in oxygen-permeable bags (polyethylene, 2,300 mL/m2/24 h, 100 mm×150 mm, total thickness 0.0508 mm), stored for 14 d at 4°C±1°C. Analyses were conducted at 0, 3, 7, and 14 days of storage (n = 3 sausages per treatment (4) per storage time (4); n = 48 samples). The analysis of each parameter was performed in three observation (total number of tested samples n = 192 for testing parameters 2-thiobarbituric acid reactive substances [TBARS], carbonyl, volatile compounds and sensory).

### Lipid oxidation

The TBARS content was determined. Diluted 1,1,3,3-tetramethoxypropane (1 mg/L) (Sigma-Aldrich, Beijing, China) was used as standard solution for determining malondialdehyde (MDA) content. Briefly, 2.5 g sausage sample was mixed with 20 mL distilled water, and then the mixture was homogenized in a high-speed homogenizer (T10 basic ULTRA, IKA, Staufen, Germany). Five milliliters of trichloroacetic acid (25%) were added to the homogenate, followed by stirring at 4°C for 15 min. The stirred liquid was centrifuged (3–30KS, Sigma, Germany) at 4°C (10,000 g, 15 min), and the supernatant was mixed with 1.5 mL of thiobarbituric acid (0.6%), and in water bath at 70°C for 30 min, then the absorbance of samples was read at 532 nm using a UV-Vis spectrophotometer (U5100, Hitachi, Tokyo, Japan). The TBARS was calculated as mg of MDA/kg of sausage.

### Protein oxidation

Protein oxidation was determined by the method described by Xiao et al [18]. Briefly, carbonyl groups were reacted with 2,4-dinitrophenylhydrazine to develop protein hydrazone. The value of reaction products was measured by a UV-Vis spectrophotometer (U5100, Hitachi, Japan) at 370 nm. The protein concentration was calculated by measuring the absorbance at 280 nm, and bovine serum albumin was used as standard. The carbonyl content was calculated as nanomoles per mg of protein, using an absorption coefficient of 22,000.

### Volatile compounds

Sausages were cut into small pieces and 3.0 g sample was put into a 20 mL headspace vial. The volatile compounds were determined using GC/MS (7890A-5975C, Agilent Technologies, Wilmington, DE, USA) with a HP-5 silica capillary column (60 m×0.32 mm×0.25 μm, Agilent Technologies, USA), and volatile compounds of sausages were extracted by headspace-solid-phase microextraction (HS-SPME, DVB/CAR/PDMS fiber, Supelco, Bellefonte, PA, USA). Prior to extraction of volatiles, the fiber was preconditioned at 230°C for 30 min in the gas chromatography (GC) injection port. The sample was extracted in headspace vail at 60°C for 15 min. The SPME fiber was inserted into the headspace vail through the septum and absorbed volatile compounds at 60°C for 30 min. The SPME fiber was desorpted 5 min in the injection port at 230°C during the chromatographic run. Helium with a flow rate of 1.0 mL/min was used as the carrier gas. The injection port was in splitless mode. The temperature program was isothermal for 15 min at 40°C, then increased to 190°C at a rate of 4°C/min, and then increased to 250°C at 10°C/min. The transfer line to the mass spectrometer was maintained at 250°C. The mass spectrometer was obtained by electronic impact at 70 eV. The data was obtained at a rate of s^−1^ over a range of 33 to 450 for m/z. And the NIST 11 mass spectral library was used to identify the volatile compounds. Results were expressed as percentage of total chromatographic area.

### Sensory evaluation

Sensory evaluation of sausage samples was performed by a group consisted of 20 panel members (10 females and 10 males), and they were trained according to the standard procedure: samples were cut into 1 cm sections, followed by randomly numbered with digit and placed in a random order, and then the samples were placed on plates and served to the panelists at 25°C. The panelists were provided 100 mL water to gargle before evaluating the sausage samples. The sensory properties of sausages were evaluated by their color, smell, taste, sourness, chewiness, and overall acceptability. A 9-point hedonic scale was used: for color, 1 = dislike very much, 5 = neither like nor dislike, 9 = like very much; for smell, 1 = very weak, 5 = neither strong nor weak, 9 = very strong; for taste, 1 = dislike, 5 = neither like nor dislike, 9 = like very much; for sourness, 1 = very weak, 5 = neither strong nor weak, 9 = very strong; and for chewiness, 1 = very tough, 5 = neither tough nor tender, 9 = very tender. And panelists also evaluated the over acceptability of sausages, 1 = dislike very much, 5 = neither like nor dislike, 9 = like very much.

### Statistical analysis

The experimental data was processed by SPSS 20.0 software (SPSS, Chicago, IL, USA). Comparison of means of values among different treatments were subjected to one-way analysis of variance, where the measured variables were set as dependent variables, different treatments (Control, BHA, AE, EEME) and four display times (0, 3, 7, and 14) as fixed effects, and replicate as random effect. For sensory evaluation data, all panelists were included in sensory evaluation portion of analysis. A mixed statistic model was created with time, treatment and panelists as fixed effects and the session was considered as random effect. When significant differences were found, differences among the means were compared in accordance with Duncan’s multiple range test, and a value of p<0.05 was considered statistically significant. The values were given in terms of means±standard error of three replicates in Tables.

## RESULTS AND DISCUSSION

### The antioxidant capacity of mushroom extract

**Figure 2:**
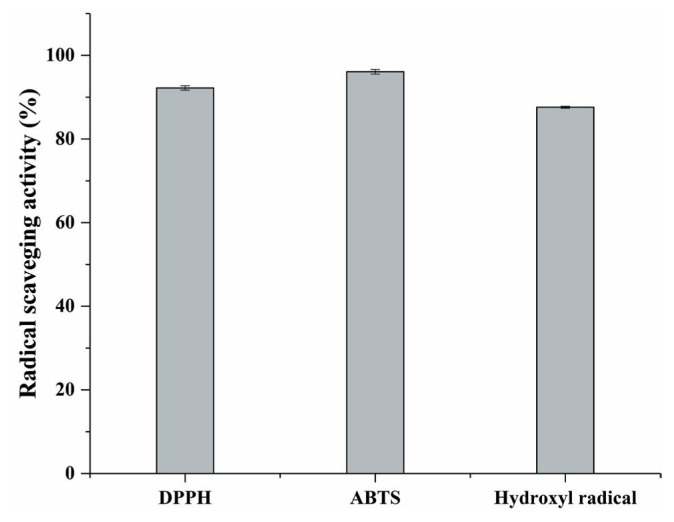
The radical scavenging activity of *Flammulina velutipes* extracts. Error bars refer to the standard error obtained from triplicate sample analysis.

Early studies have demonstrated that mushroom extract had free radical scavenging capacity and antioxidant activity *in vitro* and *in vivo* [10]. The abilities to scavenge free radicals of DPPH, ABTS and hydroxyl were widely used to test the antioxidant activity of samples against reduction/oxidation initiated by free radicals [17]. In Figure 2, it was shown that the *F. velutipes* extract revealed strong scavenging activities against free radicals DPPH (90.2%), ABTS (96%), and hydroxyl (87.6%). Based on previous studies, similar results were obtained by Bao et al [10], they found that DPPH radical scavenging rate and total reducing power rate of extract prepared from *F. velutipes* fruiting body were about 85% and 90% (volume of extracts 20 μL).

### Oxidative stability

In this study, the effects of natural antioxidant ESH-enriched mushroom extract against lipid and protein oxidation occurred in emulsified sausage were detected. The TBARS value was used to evaluate the lipid oxidation in meat system. As shown in Table 1, at day 0, 3, 7, and 14, the TBARS values of sausages from EEME group were 0.12, 0.27, 0.43, and 0.80 mg/kg sample, which were significantly lower than those in control group by 36.84%, 28.95%, 31.75%, and 34.62% at the respective storage times (p<0.05). For sausages from BHA group, the TBARS values were 0.13, 0.28, 0.48, and 0.82 mg/kg sample at day 0, 3, 7, and 14 of refrigerated storage, and no difference was found between group EEME and group BHA at each storage time. It was demonstrated that adding ESH-enriched *F. velutipes* extract (0.8%) to sausage could effectively prevent lipid oxidation during refrigerated storage, and its efficacy was equivalent to 0.01% BHA. In current study, the content of protein carbonyls was used to evaluate the degree of protein oxidation occurring in sausages. As shown in Table 2, the carbonyl values of sausages from EEME group were 0.032, 0.28, 0.76, and 0.92 nmol/mg at day 0, 3, 7, and 14 of refrigerated storage, which were significantly lower than those in control group by 33.33%, 36.36%, 25.49%, and 28.68% at the respective storage times (p<0.05). For the samples from BHA batch, at day 0, 3, 7, and 14, no difference was found between group BHA and group EEME. This data indicated that ESH-enriched *F. velutipes* extract (0.8%) also could effectively prevent protein oxidation of sausage, and its efficacy was equivalent with 0.01% BHA. The results obtained in this study agreed with previous studies conducted on raw muscle food, these studies concluded that adding ESH-enriched mushroom extract was an effective method to control the oxidative reactions occurring in tuna [9], yellowtail dark muscle and beef patties [10], shrimp [12], and salmon [13].

During meat processing, many factors could affect the antioxidative activity of natural antioxidants, such as temperature, pH, processing conditions and food additives. So, for utilization in processed meat, the antioxidants should meet many requirements, among which being stable of heat, light and acid-base is important. Liu et al [19] studied the stability of ESH from mushroom extract and found that it had excellent light, thermal and acid-base stability, this is very ubiquitous advantage for processed meat production. It is suggested that the extract of *F. velutipes* contained high-level of ESH and could be a good antioxidant candidate for meat processing.

Numerous studies have revealed that extract of certain mushrooms had strong antioxidative ability, and the active substances in the extract (extracting with water or alcohol) including ESH and certain phenolic compounds [10]. Nguyen et al [20] demonstrated that, both ESH and total phenolic compounds (TPs) showed a significant correlation with DPPH radical scavenging capacity, and the ESH had higher correlation (R^2^ = 0.84) with DPPH radical scavenging activity than TPs (R^2^ = 0.52). Pahila et al [7] also concluded that a high correlation (R^2^ = 0.9984) was found between ESH content in crude mushroom extract and DPPH radical scavenging. Meanwhile, Bao et al [10] also reported that ESH in mushrooms mainly contributed to the DPPH radical scavenging capacity. While Liu et al [19] found that, after isolating ESH from mushrooms, the antioxidant ability of ESH accounted for about 25% of the total antioxidant ability of the extract. In current study, to clarify whether ESH in *F. velutipes* extract plays a major role in antioxidation, we set up an AE group where authentic ESH (purity ≥98%) at a level of 0.12 g/kg (this amount of ESH was same as that of *F. velutipes* extract) was added to sausage. As shown in Table 1 and 2, compared to AE group, there was an increasing trend in the numerical values of TBARS and carbonyl in EEME group, but no significant difference was found between group EEME and group AE at day 0, 3, 7, and 14 refrigerated storage. These results confirmed that ESH mainly contributed to the antioxidative activity of *F. velutipes* extract.

As shown in Figure 1, chemically, ESH is an unusual thiol-histidine betaine with a sulfur atom linked to position 2 of imidazole ring [3,6]. It has distinctive features that are markedly different from ordinary thiols like GSH, for example, under physiological pH, ESH does not oxidize automatically as rapidly as GSH, and it does not promote the production of hydroxyl radicals by H_2_O_2_ and Fe^2+^ ions [21]. Another important characteristic of ESH is that the standard redox potential of thiol-disulfide couple of ESH at pH 7 is −0.06 V, while other widely occurring natural thiols typically range from −0.20 to −0.32 V [22]. Because of these excellent characteristics, ESH revealed strong antioxidant capacity to control oxidative damage.

In the process of meat production, various physical operations (such as grinding, cooking, emulsification, and deboning) can cause muscle membrane system disruption, which is conducive to the oxidation reaction in meat. It is well accepted that polyunsaturated fatty acids in meat are beneficial to oxidation because the double bond in polyunsaturated fatty acids is an ideal initiator in the oxidation process, it reacts with oxygen and other fatty acids in the atmosphere to form hydrogen peroxide and free radicals [2,23]. This process continues until production of final products, such as aldehydes, ketones, and hexanes, which cannot further support the oxidation cycle [23]. Meanwhile, lipid oxidation and free radicals could promote protein oxidation occurring in meat and result in protein carbonylation, polymerization and coagulation [24]. In addition to the composition and content of lipids and proteins in meat, the oxidation process is also affected by other factors, such as light, heat, metal ions, heme pigments, low pH, oxidative enzymes, sodium chloride and other ingredients [2,23]. So, the substances which could act as free radical terminators (e.g. BHA and BHT), free radical preventors (e.g. metal complexing agents ethylenediaminetetraacetic acid) and redox compounds (e.g. cysteine and ascorbic acid) could retard the oxidative damage in meat [23]. Many researchers did good work on antioxidative mechanism of ESH, they demonstrated that ESH is an active scavenger of free radicals, including H_2_O_2_, •OH, ^1^O_2_, lipid peroxides, nitric oxide derivatives and superoxide ion [3]. Meanwhile, earlier studies discovered that ESH could chelate divalent metal cations, including Cu^2+^, Hg^2+^, Zn^2+^, Cd^2+^, Co^2+^, Fe^2+^, and Ni^2+^ [25], and the Cu^2+^ could form a most stable complex with ESH in the form at a molar ratio of 2:1 of ESH to metal ion [25]. In the meat system, the ability of binding these metal ions could help to decrease the generation of reactive oxygen species (ROS). Encarnacion et al [12], that ESH interacted directly with Cu^2+^ at the putative binding sites of polyphenoloxidase, based on this reaction, ESH could prevent shrimp from melanosis. Therefore, there is a strong possibility that, during emulsified sausage processing and storage, ESH exerts the antioxidant activities through donating hydrogen to the free radicals and controlling the production of ROS through chelating metal ions, acting alone or in combination. While further studies are needed to better understand the antioxidant mechanism of ESH in meat system.

### Volatile compounds

It is established that flavor is one of the most important quality attributes contributing to the eating quality of meat. In current study, using SPME-GC-MS, 36 volatile compounds were identified during the storage of emulsified sausage (Table 3), they were classified into 6 groups including aldehydes (6), hydrocarbons (10), ketones (2), alcohols (1), esters (14), and aromatics (3). Among them, aldehydes were the main component of which hexanal dominated. As described by previous work, the level of off-flavor can be an ultimate factor determining consumer purchases [23], and the lipid oxidation in meat matrix was associated with off-flavor [26], furthermore, it was shown that the hexanal content was widely used as indicator of lipid oxidation in meat system [26]. As shown in Table 3, at day 0, 3, 7, and 14, the concentrations of hexanal in sausages from groups BHA, AE, and EEME were significantly (p<0.05) lower than those from control group, while, no significant difference was found among groups BHA, AE, and EEME. In terms with pentanal, at day 0, 3, 7, and 14, the concentrations of pentanal in sausages from groups BHA, AE, and EEME were significantly (p<0.05) lower than those from control group. These results were in line with the findings of Frankel and Tappel [27], who concluded that the amounts of pentanal correlated well with the corresponding amounts of hexanal in meat. These data confirmed our above observations in lipid oxidation measured by TBARS, and they were also consistent with sensory evaluation of sausage.

Volatile hydrocarbons have a relatively high aroma threshold [28], so, they may not have a major impact on meat flavor, and there is evidence that volatile hydrocarbons are produced during lipid peroxidation [23]. In present study, at day 0, 3, 7, and 14, the total hydrocarbon amounts in sausages from EEME group was significantly (p<0.05, Table 3) lower than other groups. These results agreed with the TBARS values shown in Table 1. It is reported that volatile alcohols are usually associated with fresh, fruity and fatty aromas [29], while the aroma threshold of alcohols is higher than that of aldehydes and ketones [30]. In this study, large amounts of ethanol were found in all groups, but there was no difference among groups control, BHA, AE, and EEME. The alcohol-derived volatiles in sausages should be related to the wine added during manufacturing. It is described that most of volatile ketones have milky and fruity aroma [16], while they poorly contribute to the flavor of sausage due to the high aroma threshold value. In current study, only two kinds of ketones were detected, they were 2,3-octanedione and acetophenone. As shown in Table 3, at day 3, 7, and 14, the 2,3-octanedione amounts in sausages from groups BHA, AE, and EEME were significantly (p<0.05) lower than those from control group. Based on previous studies, ketones are another major product of lipid oxidation, accordingly, these results coincided with the results of lipid oxidation as shown in Table 1. From Table 3, it is shown that, at day 0 and 3, higher amounts of 2-pentyl-furan were detected in EEME group. It is reported that many kinds of mushroom contained 2-pentyl-furan which can provide vegetable-like, earthy and bean aroma [31]. Therefore, the higher levels of 2-pentyl-furan identified in sausages of EEME group could be derived from mushroom extract. While, at day 7 and 14, the amounts of 2-pentyl-furan in EEME group were lower than control group. As discussed above, this phenomenon can be explained by the lower oxidative reaction occurred in sausages from EEME group, because 2-pentyl furan could be formed from deterioration of lipid and protein at low temperature storage [31].

It is demonstrated that volatile esters are generally responsible for fruity and flowery smell of food [16], and they are formed by the interaction of alcohol and free fatty acid produced by lipid oxidation [32]. During storage of emulsified sausages, 14 kinds of ester compounds were identified in samples from EEME group (Table 3), and they were dominated by hexanoic acid, ethyl ester (Table 3), this phenomenon could be attributed to ester compounds contained in mushroom extract [33], or these ester compounds were formed in other reactions taking place in stored sausage. Overall, above data demonstrated that adding ESH-enriched mushroom extract to sausage could be able to enrich flavor composition and increase content of characteristic flavor of emulsified sausage.

### Sensory evaluation

**Figure 3:**
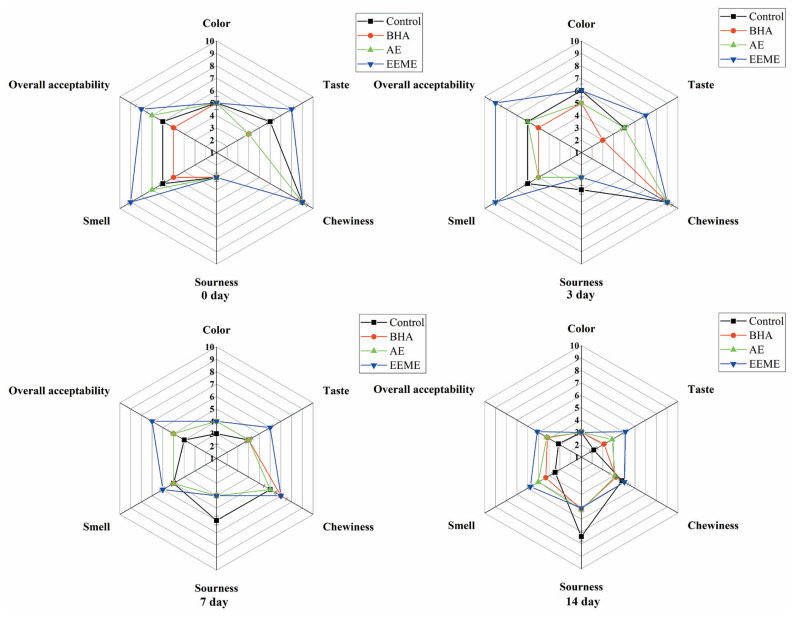
Sensory properties of emulsified sausage during refrigerated storage treated with antioxidants BHA, AE, and EEME. Control, no antioxidant; EEME, with 0.8% ergothioneine (ESH)-enriched *Flammulina velutipes* extracts; AE, with 0.012% authentic ergothioneine (ESH); BHA, BHA with 0.01% butylated hydroxyanisole.

The effects of adding BHA, AE, and EEME on the sensory characteristics of emulsified sausages were studied. The sensory parameters including color, taste, chewiness, smell, sourness, and overall acceptability of sausages were assessed. As shown in Figure 3, compared to other groups, the sausages in EEME group got the highest scores in taste and smell at day 0, 3, 7, and 14 refrigerated storage, this may be due to the “good components” contained in mushroom extract, such as phenolic, flavonoid and free amino acids [34], which can promote the taste and flavor properties of sausages. As described above, adding mushroom extract to sausage could effectively prevent lipid oxidation and improve flavor attributes of emulsified sausage during refrigerated storage. Therefore, these results obtained from sensory evaluation coincided with the findings from oxidative stability and volatile compounds. While, for parameters of color and chewiness, no significant difference was found among four groups. Sourness taste could be an indicator of rancid for emulsified sausages, at day 3, 7, and 14 refrigerated storage, the sourness score of sausages from control group was higher than those from groups BHA, AE, and EEME. This indicates that the quality of sausages without adding any antioxidant significantly decreased with storage time increased.

## CONCLUSION

The *F. velutipes* extract has high-level of ESH (15.29±0.52 mg/g extract), it produced excellent scavenging activities against free radicals DPPH (90.2%), ABTS (96%), and hydroxyl (87.6%). It was demonstrated that adding ESH-enriched *F. velutipes* extract to sausage could effectively prevent lipid and protein oxidation during refrigerated storage, and its efficacy was equivalent to 0.01% BHA. During meat processing, the ESH mainly contributed to the antioxidative activity of *F. velutipes* extract. The flavor and sensory attributes of emulsified sausage could be improved through adding ESH-enriched *F. velutipes* extract. In conclusion, the extract of *F. velutipes* contained high-level of ESH could be a good natural antioxidant candidate in processed meat production.

## Figures and Tables

**Table 1:** Effect of ergothioneine-enriched mushroom extract on the TBARS content of emulsified sausage during refrigerated storage

Group^1)^	TBARS (mg MDA/kg sample)			

	0 d	3 d	7 d	14 d
Control	0.19±0.01^a^	0.38±0.03^a^	0.63±0.05^a^	1.23±0.08^a^
BHA	0.13±0.01^b^	0.28±0.01^b^	0.48±0.02^b^	0.82±0.06^b^
AE	0.15±0.02^a^	0.30±0.02^b^	0.51±0.05^b^	0.87±0.12^b^
EEME	0.12±0.01^b^	0.31±0.01^b^	0.43±0.04^b^	0.80±0.04^b^

The values are expressed as mean±standard deviation.

TBARS, 2-thiobarbituric acid reactive substances; MDA, malondialdehyde.

1) Control, no antioxidant; BHA, BHA with 0.01% butylated hydroxyanisole; AE, with 0.012% authentic ergothioneine (ESH); EEME, with 0.8% ergothioneine (ESH)-enriched *Flammulina velutipes* extracts.

a,b Means with different letters in the same column are different (p<0.05).

**Table 2:** Effect of ergothioneine-enriched mushroom extract on the protein carbonyls content of emulsified sausage during refrigerated storage

Group^1)^	Carbonyls (nmol/mg protein)			

	0 d	3 d	7 d	14 d
Control	0.048±0.01^a^	0.44±0.06^a^	1.02±0.02^a^	1.29±0.01^a^
BHA	0.037±0.03^b^	0.27±0.02^b^	0.84±0.05^b^	0.95±0.10^b^
AE	0.040±0.05^b^	0.31±0.06^b^	0.83±0.02^b^	0.96±0.02^b^
EEME	0.032±0.02^b^	0.28±0.01^b^	0.76±0.04^b^	0.92±0.03^b^

The values are expressed as mean±standard deviation.

1) Control, no antioxidant; BHA, BHA with 0.01% butylated hydroxyanisole; AE, with 0.012% authentic ergothioneine (ESH); EEME, with 0.8% ergothioneine (ESH)-enriched *Flammulina velutipes* extracts.

a,b Means with different letters in the same column are different (p<0.05).

**Table 3:** Effect of ergothioneine-enriched mushroom extract on the volatile compounds of emulsified sausage during refrigerated storage

Volatile compounds	0 d				3 d				7 d				14 d			
			
	Control^1)^	BHA^1)^	AE^1)^	EEME^1)^	Control	BHA	AE	EEME	Control	BHA	AE	EEME	Control	BHA	AE	EEME
Decane	ND	ND	ND	ND	0.90^c^	3.25^a^	3.15^a^	1.83^b^	0.14^b^	2.12^a^	2.13^a^	2.2^a^	0.47^b^	1.95^a^	1.97^a^	1.84^a^
Undecane	ND	ND	ND	ND	7.21^a^	7.96^a^	7.19^a^	4.54^b^	10.24^a^	8.17^b^	8.61^b^	5.19^c^	14.98^a^	9.44^b^	9.37^b^	7.32^c^
Pentadecane	ND	ND	ND	ND	1.46^a^	ND^b^	ND^b^	ND^b^	1.58^a^	ND^b^	ND^b^	0.15^b^	ND	ND	ND	ND
Heptadecane	ND	ND	ND	ND	ND	ND	ND	0.68	ND	ND	ND	ND	ND	ND	ND	ND
Dodecane	0.82^b^	0.73^b^	1.07^a^	0.75^b^	ND	ND	ND	ND	ND	ND	ND	ND	ND	ND	ND	ND
Tetradecane	1.20^a^	0.84^b^	0.89^b^	0.51^c^	ND	ND	ND	ND	0.26	ND	ND	ND	ND	ND	ND	ND
Pentane	ND	ND	ND	ND	ND	ND	ND	ND	ND^b^	ND^b^	ND^b^	0.61^a^	ND	ND	ND	ND
Octane	1.05^a^	NDb	ND^b^	ND^b^	ND	ND	ND	ND	ND	ND	ND	ND	ND	ND	ND	ND
**Total hydrocarbons**	**3.07** ^a^	**1.57** ^b^	**1.96** ^b^	**1.27** ^c^	**9.57** ^a^	**11.21** ^a^	**10.34** ^a^	**6.37** ^b^	**12.22** ^a^	**10.29** ^b^	**10.74** ^b^	**8.15** ^c^	**15.45** ^a^	**11.39** ^b^	**11.34** ^b^	**9.16** ^c^
Pentanal	1.84^a^	ND^c^	0.98^b^	1.21^b^	2.27^a^	ND^c^	1.46^b^	1.29^b^	2.01^a^	ND^c^	0.53^b^	0.39^b^	2.51^a^	ND^c^	1.72^b^	1.67^b^
Hexanal	30.97^a^	11.4^b^	12.05^b^	11.57^b^	31.91^a^	16.5^b^	18.28^b^	15.47^c^	32.6^a^	20.75^b^	22.39^b^	18.06^b^	34.13^a^	24.22^b^	25.13^b^	22.37^b^
Octanal	1.52^a^	1.56^a^	2.05^a^	0.99^b^	1.90^a^	2.31^a^	2.08^a^	1.76^b^	2.88^a^	2.68^a^	2.66^a^	1.85^b^	3.59^a^	2.87^b^	2.79^b^	2.47^b^
Nonanal	13.99^a^	9.67^c^	10.23^b^	11.44^b^	9.36^a^	6.97^b^	9.46^a^	10.78^a^	8.38^a^	7.04^c^	8.61^a^	7.81^b^	6.67^a^	4.66^b^	6.47^a^	4.38^b^
Benzaldehyde	0.7^b^	1.10^a^	1.22^a^	1.17^a^	1.42	1.59	1.79	1.44	ND	ND	ND	0.99	ND	ND	ND	0.39
2-Octenal	ND	ND	ND	ND	ND	ND	ND	0.09	0.77	ND	0.62	1.08	ND	ND	ND	ND
**Total aldehydes**	**49.02** ^a^	**23.73** ^b^	**26.54** ^b^	**26.16** ^b^	**46.86** ^a^	**27.36** ^c^	**33.06** bc	**30.82** ^c^	**46.64** ^a^	**30.48** ^b^	**34.81** ^b^	**30.16** ^b^	**46.90** ^a^	**31.75** ^b^	**36.11** ^b^	**31.28** ^b^
2,3-Octanedione	1.31^a^	0.38^b^	1.34^a^	1.24^a^	1.69^a^	0.71^c^	1.06^b^	1.26^b^	1.64^a^	0.69^b^	0.94^b^	0.64^b^	1.84^a^	1.24^b^	1.33^b^	1.18^b^
Acetophenone	ND	ND	ND	ND	ND	ND	1.19	ND	0.81	0.82	1.06	1.06	0.32	0.51	0.47	0.58
**Total ketones**	**1.31** ^a^	**0.38** ^b^	**1.34** ^a^	**1.24** ^a^	**1.69** ^a^	**0.71** ^c^	**2.25** ^a^	**1.26** ^b^	**2.45** ^a^	**1.51** ^b^	**2.00** ^b^	**1.70** ^b^	**2.16** ^a^	**1.75** ^b^	**1.80** ^b^	**1.76** ^b^
Ethanol	15.54	15.36	15.79	15.63	15.32	15.13	15.75	15.67	15.37	15.65	15.83	15.81	15.48	15.47	15.78	15.64
**Total alcohols**	**15.54**	**15.36**	**15.79**	**15.63**	**15.32**	**15.13**	**15.75**	**15.67**	**15.37**	**15.65**	**15.83**	**15.81**	**15.48**	**15.47**	**15.78**	**15.64**
Ethyl acetate	2.42^a^	ND^b^	3.23^a^	2.58^a^	2.35^a^	ND^c^	1.56^b^	2.27^a^	2.11^a^	ND^b^	1.94^a^	1.99^a^	1.41^b^	ND^c^	1.57^b^	1.82^a^
Hexanoic acid, ethyl ester	12.75^c^	12.13^c^	19.25^b^	29.64^a^	12.40^b^	6.31^c^	22.12^a^	25.29^a^	13.65^b^	3.78^c^	19.98^b^	24.74^a^	13.37^b^	3.01^c^	18.01^b^	23.03^a^
Octanoic acid, ethyl ester	1.5^ab^	1.25^b^	2.09^a^	1.76^a^	1.61^a^	1.21^b^	1.75^a^	1.73^a^	0.99^b^	0.90^b^	1.34^a^	1.37^a^	0.16^b^	0.19^b^	0.63^a^	0.60^a^
2,4-Hexadienoic acid, ethyl ester	3.97^ab^	3.75^b^	5.87^a^	4.98^a^	4.02^a^	3.31^b^	4.28^a^	4.69^a^	2.64^b^	2.92^b^	3.87^a^	3.68^a^	2.46^b^	2.61^b^	3.68^a^	3.54^a^
Benzoic acid, ethyl ester	2.13^b^	1.63^c^	2.93^a^	3.00^a^	1.72^a^	ND^c^	1.58^b^	1.47^b^	1.08^a^	0.42^c^	0.71^b^	0.81^ab^	0.85^a^	0.55^b^	0.33^c^	0.37^c^
2,2,4-Ttrimethyl-3-Pentanoicacid-carboxyisopropyl, isobutyl ester	4.14^c^	4.76^b^	8.8^a^	7.74^a^	ND	ND	ND	ND	ND	ND	ND	ND	ND	ND	ND	ND
Phthalic acid, allyl ethyl ester	1.26^a^	ND^b^	ND^b^	ND^b^	0.07	ND	ND	ND	ND	ND	ND	ND	ND	ND	ND	ND
Dibutyl phthalate	1.54^a^	0.78^b^	1.97^a^	1.79^a^	0.82	0.62	0.47	0.72	0.29	ND	ND	ND	0.14	ND	ND	ND
Hexanedioic acid, bis (2-methylpropyl) ester	0.06	0.04	ND	ND	0.21	0.06	ND	ND	ND	ND	ND	ND	ND	ND	ND	ND
2-Methyl-,1-(1,1-dimethylethyl)-1,3-propanediyl ester	ND	0.06	ND	ND	0.07	0.35	0.42	0.41	0.06	0.4	0.51	0.4	ND	ND	ND	ND
Adipic acid, isobutyl 2-methylpent-3-yl ester	ND^b^	1.00^a^	1.32^a^	1.16^a^	ND^c^	ND^c^	1.36^b^	1.90^a^	ND^c^	ND^c^	1.82^b^	2.44^a^	ND^c^	ND^c^	2.64^b^	3.41^a^
Heptanoic acid, ethyl ester	ND^b^	ND^b^	0.46^b^	1.21^a^	ND^b^	ND^b^	0.82^a^	1.34^a^	ND^b^	ND^b^	1.31^a^	1.33^a^	ND^c^	ND^c^	2.39^b^	2.98^a^
2-Ethylhexyl acrylate	ND	ND	ND	ND	2.14^a^	ND^b^	2.95^a^	3.2^a^	0.95^d^	1.17^c^	2.07^b^	5.63^a^	ND^d^	1.51^c^	3.57^b^	4.65^a^
Butanoic acid, ethyl ester	ND	ND	ND	ND	ND	0.31	0.35	ND	0.08^b^	ND^b^	0.66^a^	0.94^a^	ND	ND	ND	ND
**Total esters**	**29.78** ^c^	**25.4** ^d^	**45.92** ^b^	**53.85** ^a^	**25.4** ^c^	**12.17** ^d^	**37.64** ^b^	**43.03** ^a^	**21.85** ^c^	**9.59** ^d^	**34.21** ^b^	**43.32** ^a^	**18.39** ^c^	**7.87** ^d^	**32.82** ^b^	**40.40** ^a^
Butylated hydroxytoluene	ND^b^	31.81^a^	ND^b^	ND^b^	ND^b^	31.42^a^	ND^b^	ND^b^	ND^b^	30.60^a^	ND^b^	ND^b^	ND^b^	30.31^a^	ND^b^	ND^b^
Toluene	ND	ND	ND	ND	ND	ND	ND	ND	ND^b^	1.40^a^	1.60^a^	ND^b^	ND^b^	1.23^b^	1.49^a^	ND^b^
2-Pentyl-furan	1.28^b^	1.74^a^	1.96^a^	1.85^a^	1.16^b^	ND^c^	0.95^b^	1.85^a^	1.46^a^	0.48^c^	0.81^b^	0.85^b^	1.62^a^	0.23^c^	0.66^b^	0.76^b^
**Total aromatics**	**1.28** ^b^	**33.56** ^a^	**1.96** ^b^	**1.85** ^b^	**1.16** ^b^	**31.42** ^a^	**0.95** ^b^	**1.85** ^b^	**1.46** ^b^	**32.48** ^a^	**2.41** ^b^	**0.85** ^b^	**1.62** ^b^	**31.77** ^a^	**2.15** ^b^	**0.76** ^b^

Volatile compounds expressed as % of total peak area.

ND, not detected.

1) Control: no antioxidant; BHA, BHA with 0.01% butylated hydroxyanisole; AE, with 0.012% authentic ergothioneine (ESH); EEME, with 0.8% ergothioneine (ESH)-enriched *Flammulina velutipes* extracts.

a–d Means with different letters superscript in the same row are significant different (p<0.05).
